# Intralesional TLR4 agonist treatment strengthens the organ defense against colonizing cancer cells in the brain

**DOI:** 10.1038/s41388-022-02496-3

**Published:** 2022-10-12

**Authors:** Raquel Blazquez, Han-Ning Chuang, Britta Wenske, Laura Trigueros, Darius Wlochowitz, Renato Liguori, Fulvia Ferrazzi, Tommy Regen, Martin A. Proescholdt, Veit Rohde, Markus J. Riemenschneider, Christine Stadelmann, Annalen Bleckmann, Tim Beißbarth, Denise van Rossum, Uwe K. Hanisch, Tobias Pukrop

**Affiliations:** 1 Department of Internal Medicine III, Hematology and Medical Oncology, University Hospital Regensburg, 93053 Regensburg, Germany; 2 Department of Hematology and Medical Oncology, University Medical Center Göttingen, 37075 Göttingen, Germany; 3 Institute of Medical Bioinformatics, University Medical Center Göttingen, Georg August University, 37075 Göttingen, Germany; 4 Institute of Pathology, Friedrich-Alexander-Universität Erlangen-Nürnberg, 91054 Erlangen, Germany; 5 Department of Nephropathology, Institute of Pathology, Friedrich-Alexander-Universität Erlangen-Nürnberg, 91054 Erlangen, Germany; 6 Institute of Neuropathology, University Medical Center Göttingen, 37075 Göttingen, Germany; 7 University Medical Center of the Johannes Gutenberg University, 55131 Mainz, Germany; 8 Department of Neurosurgery, University Hospital Regensburg, 93053 Regensburg, Germany; 9 Department of Neurosurgery, University Medical Center Göttingen, 37075 Göttingen, Germany; 10 Department of Neuropathology, University Hospital Regensburg, 93053 Regensburg, Germany; 11 Department of Medicine A, Hematology, Oncology and Pneumology, University Hospital Münster, 48149 Münster, Germany; 12 Sartorius Canada Inc., Oakville, ON L6M 2V9 Canada; 13 Division of Personalized Tumor Therapy, Fraunhofer Institute for Toxicology and Experimental Medicine, 93053 Regensburg, Germany

**Keywords:** Metastasis, Tumour immunology

## Abstract

Brain metastasis in breast cancer remains difficult to treat and its incidence is increasing. Therefore, the development of new therapies is of utmost clinical relevance. Recently, toll-like receptor (TLR) 4 was correlated with IL6 expression and poor prognosis in 1 215 breast cancer primaries. In contrast, we demonstrated that TLR4 stimulation reduces microglia-assisted breast cancer cell invasion. However, the expression, prognostic value, or therapeutic potential of TLR signaling in breast cancer brain metastasis have not been investigated. We thus tested the prognostic value of various TLRs in two brain-metastasis gene sets. Furthermore, we investigated different TLR agonists, as well as MyD88 and TRIF-deficient microenvironments in organotypic brain-slice ex vivo co-cultures and in vivo colonization experiments. These experiments underline the ambiguous roles of TLR4, its adapter MyD88, and the target nitric oxide (NO) during brain colonization. Moreover, analysis of the gene expression datasets of breast cancer brain metastasis patients revealed associations of TLR1 and IL6 with poor overall survival. Finally, our finding that a single LPS application at the onset of colonization shapes the later microglia/macrophage reaction at the macro-metastasis brain-parenchyma interface (MMPI) and reduces metastatic infiltration into the brain parenchyma may prove useful in immunotherapeutic considerations.

## Introduction

Brain metastasis (BM) occurs frequently in lung and breast carcinomas [1, 2]. The prevalence is increasing and the efficacy of current therapies remains limited, making BM especially from breast cancer (BCBM) a lethal disease with low survival [3]. Moreover, the brain is inherently the most immune-suppressed organ, such that inflammatory pathways are also less activated in BM. Thus, it is all the more surprising that the toll-like receptor (TLR) signaling pathway is upregulated in brain metastasis of lung-cancer patients [4] and that TLR4 is expressed in microglia during colonization of the brain in a lung-cancer model [5]. However, the role of TLR signaling in BCBM has not been investigated yet. Nevertheless, in breast primary tumors, TLRs are downregulated in general, but TLR4 expression is associated with poor prognosis [6].

TLRs recognize exogenous pathogen-associated molecular patterns (PAMPs), as well as endogenous ligands, called damage-associated molecular patterns (DAMPs) [7]. In humans, ten TLRs have been identified. TLR1, 2, 4, 5, 6, and 10 are mainly expressed on the plasma membrane, where they recognize bacterial components, such as lipoprotein of Gram-positive bacteria (TLR1, 2, 6, and 10), lipopolysaccharide (LPS) of Gram-negative bacteria (TLR4), and flagellin of bacterial flagella (TLR5). TLR3, 7, 8, and 9 are located intracellularly, and are mostly able to detect bacterial or viral nucleic acids, like double-stranded RNA/polyinosinic–polycytidylic acid [poly(I:C)] (TLR3), single-stranded RNA (TLR7), or CpG-containing DNA (CpG-ODN) (TLR9) [8, 9].

In cancer, dying or necrotic tumor cells, damaged extracellular matrix (ECM), or leaky blood vessels release multiple DAMPs, activating their respective receptors. Thus, numerous DAMPs and their combinations are present during cancer progression. For example, TLR4 is activated by ECM components like tenascin C or fibronectin; by plasma membrane components like syndecan or gylpican; and intracellular molecules like HMGB1, S100 proteins, or histones, to name just a few. Most importantly, DAMP binding to TLRs often leads to different signaling outcomes than PAMP ligands, both quantitatively and qualitatively [10, 11].

Upon activation, TLRs transmit signals through one or more adapter proteins. All TLRs (except for TLR3) signal through at least MyD88. TLR3 signals through the TRIF pathway; TLR4 signals through both adapters [9]. Additionally, TLR stimulation can lead to the activation of several pathways in parallel, such as NF-κB, MAPKs, Jun N-terminal kinases, p38, as well as interferon regulatory factors 3, 5, and 7. These potential multiple pathway activations result in a plethora of combinations and outcomes. Moreover, TLR signaling triggers multiple biological actions, including cytokine release, gene induction, and secondary translational modifications. Thus, the outcome of TLR activation is variable and dependent on the ligands, context, and cell type [9, 12].

Despite these uncertainties, several TLR agonists are currently undergoing investigation in clinical trials. For example, the synthetic TLR4 agonist glucopyranosyl lipid A (GLA-SE) is effective against recurrent soft-tissue sarcoma expressing the NY-ESO-1 tumor antigen (CMB305) [13], and intra-tumoral GLA-SE treatment is also under investigation in Merkel cell carcinoma [14].

In previous studies, we demonstrated that stimulating TLR4 with LPS reduces microglia-assisted breast-cancer-cell invasion and that the myeloid compartment of BM can be re-educated towards a more anti-metastatic phenotype in breast-cancer colonization models [15, 16]. Basing on these previous observations, we investigated the prognostic value of various TLRs in BM tissues, the potency to push microglia/macrophages into a more anti-metastatic phenotype during CNS colonization, and aimed to rule out potential therapeutic strategies.

## Results

### Clinical relevance of TLR signaling in BCBM

**Fig. 1:**
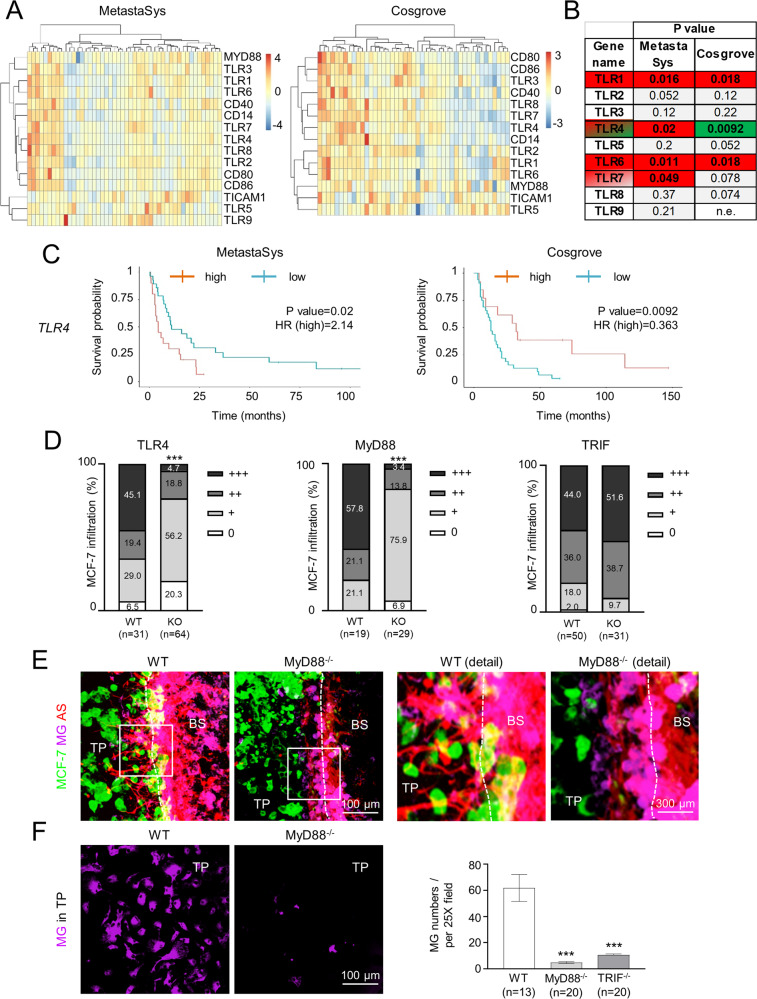
Clinical relevance of TLR expression in human brain metastases and contribution of TLR4 to tumor cell infiltration. **A** Expression heatmap of selected TLR-related genes in BCBMs patients. Unsupervised hierarchical clustering heatmap of the expression of selected genes in BCBMs MetastaSys (*n* = 48; left) and Cosgrove (*n* = 45; right) cohorts. The color code indicates gene expression (red = high; blue = low). **B** Summary table of log-rank test p-values with color code indicating the relationship between the gene expression of the indicated TLR signaling components (red = high; green = low; grey = not significant; n.e. = not expressed) and BCBM patient poorer prognosis in the BCBM patient cohorts. **C** Kaplan-Meier survival curves of BCBM patients in MetastaSys (left) or Cosgrove (right) cohorts stratified on the basis of *TLR4* expression (*P* value = log-rank test *p*-value; HR = hazard ratio). **D** Organotypic brain-slice ex vivo co-culture with MCF-7 tumor cells and brain slices from C57BL/6 wild-type (WT) and TLR4 knockout (KO), MyD88 KO, or TRIF KO mice. Data represent the percentage degree of tumor-cell infiltration into the brain slices after 96 hours (*n* ≥ 19; ***p < 0.001; Mann-Whitney test). **E** Representative pictures of MCF-7 cells co-cultured with brain slices from WT and MyD88^−/−^ mice (TP tumor plug, BS brain slice). MCF-7 tumor cells are depicted in green; activated MG (IL-B4) and AS (GFAP) are illustrated in purple and red, respectively. Images of the MMPI at higher magnification are portrayed (upper row, right). **F** Representative pictures (left) and quantification (right) of MG (IL-B4) present in the tumor plug (TP), indicated as the number of MG cells per 25X field (*n* ≥ 13; mean ± SEM; ***p < 0. 001; unpaired *t*-test).

To investigate the potential clinical relevance of TLR signaling in BCBM, we first checked the expression status of different components of the TLR signaling pathway in two BCBM patient cohorts. Heatmaps confirmed differential TLR activation in BCBM (Fig. 1A). High *TLR1* and *TLR6* expression significantly correlated with a poorer prognosis in both cohorts (Fig. 1B, Supplementary Figure S1A, and Supplementary Table 1). *TLR4* and *TLR7* also correlated with a significantly poorer prognosis in the MetastaSys cohort (Fig. 1B, C and Supplementary Table 1), as previously demonstrated for breast cancer primaries [6]. Interestingly, *TLR4* expression was associated with a significantly improved prognosis in the Cosgrove cohort (Fig. 1B, C and Supplementary Table 1). Moreover, expression of the two TLR4 adapters *MyD88* and *TICAM1* (**=***TRIF*) also correlated with opposite outcomes in both cohorts (Supplementary Fig. S1A and Supplementary Table 1). Thus, we aimed to elucidate further the role of TLR4 and its downstream adapters MyD88 and TRIF during metastatic brain colonization.

### TLR4 mediates metastatic brain infiltration via MyD88

Previously, we reported that stimulation with the TLR4 agonist LPS reduced microglia (MG)-induced invasion of MCF-7 human breast-cancer cells in a modified Boyden chamber assay [15]. Here we focused on the effect of TLR4 signaling during metastatic brain colonization. To this effect, we first tested the infiltration capacity of MCF-7 cells into TLR4-, MyD88-, and TRIF-knockout (KO) brain slices in a 3D organotypic ex vivo co-culture [17]. Unexpectedly, MCF-7 infiltration was significantly reduced in TLR4- and MyD88-deficient brain slices, but remained unchanged in TRIF-deficient brain slices (Fig. 1D). The activation of microglia (MG) and astrocytes (AS) was, however, reduced in both MyD88^−/−^ and TRIF^−/−^ brain slices, and fewer microglial cells moved into the tumor plug compared to the wild-type (WT) co-cultures (Fig. 1E, F and Supplementary Fig. S1B). These results indicate that both TLR4 adapters MyD88 and TRIF are involved in the activation of the MG-AS communication loop during the initial steps of astrogliosis. However, only MyD88 supports tumor infiltration into the brain slice. On the one hand, this result is in contradiction with our earlier observation that TLR4 activation reduces MG-induced invasion [15]. On the other hand, it supports our previous finding that colonizing metastatic cells misuse DAMP signaling in the brain parenchyma during metastatic infiltration [18].

### TLR4 activation plays an ambivalent role during MG-induced tumor cell infiltration

**Fig. 2:**
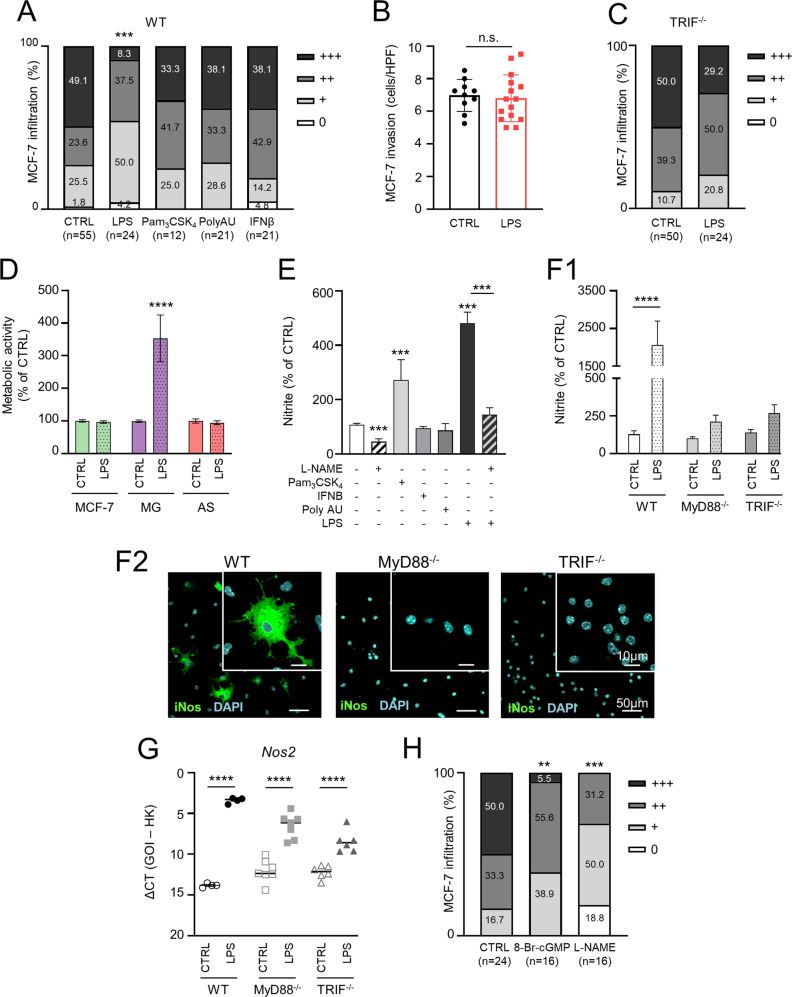
Role of MyD88, TRIF and NO in tumor cell infiltration. **A** Organotypic brain-slice ex vivo co-culture with MCF-7 tumor cells and WT brain slices stimulated with LPS (1 µg/ml), Pam_3_CSK_4_ (1 µg/ml), recombinant IFNβ (100 ng/ml), or Poly(A:U) (1 µg/ml). Data represent the percentage degree of tumor-cell infiltration into the brain slices after 96 h (*n* ≥ 12; ***p < 0.001; Mann-Whitney test). **B** MCF-7 tumor-cell invasion after PBS (CTRL) or LPS (1 µg/ml) stimulation. Data represent the average tumor cell number migrated per high power field (HPF) after 96 hours (*n* ≥ 10; mean ± SD; n.s. = not significant; unpaired *t*-test). **C** Organotypic brain-slice ex vivo co-culture with MCF-7 tumor cells and brain slices from TRIF^-/-^ mice after PBS (CTRL) or LPS (1 µg/ml) stimulation. Data represent the percentage degree of tumor-cell infiltration into the brain slices after 96 h (*n* ≥ 24; Mann-Whitney test). **D** Metabolic activity of the indicated cell types after stimulation with PBS (CTRL) or LPS (1 µg/ml) assessed by means of MTT conversion assay (*n* ≥ 8; mean ± SD; ***p < 0.001; one-way ANOVA followed by Sidak´s multiple comparisons test). **E** NO release as a function of nitrite accumulation in supernatants of WT MG after stimulation with L-NAME (1 mM), Pam_3_CSK_4_ (10 ng/ml), recombinant IFNβ (10 ng/ml), Poly(A:U) (1 µg/ml), and LPS (100 ng/ml) (*n* ≥ 6; mean ± SEM; ***p ≤ 0.001; unpaired *t*-test). **F** NO release as a function of nitrite accumulation in supernatants of WT, MyD88^−/−^, and TRIF^−/−^ MG after LPS (100 ng/ml) stimulation. (F1) NO release is indicated as the percentage of nitrite accumulated compared to the control (CTRL; *n* ≥ 5; mean ± SEM; ****p < 0.0001; one-way ANOVA followed by Sidak’s multiple comparisons). (F2) Representative confocal images depicting iNos induction after LPS stimulation. **G** Quantitative RT-PCR analysis of *Nos2* expression in wild-type (WT) or MyD88^−/−^ MG after LPS (100 ng/ml) stimulation. Data represent the ΔCt value between the gene of interest (GOI = *Nos2*) and two housekeeping (HK) genes (*n* ≥ 4; mean; ****p < 0.0001; one-way ANOVA followed by Sidak’s multiple comparisons). **H** Organotypic brain-slice ex vivo co-culture with MCF-7 tumor cells and stimulated with 8-Br-cGMP (10 µM) or L-NAME (100 µM). Data represent the percentage degree of tumor-cell infiltration into the brain slices after 96 hours (*n* ≥ 16; **p < 0.01, ***p < 0.001; Mann-Whitney test).

Intrigued by the potentially contradictory results, we next sought to analyze how TLR stimulation affects metastatic brain infiltration. For this, we treated 3D organotypic ex vivo co-cultures with the TLR4 agonist LPS, the TLR2/1 ligand Pam_3_CSK_4_, the TLR3 agonist Poly(A:U), or the TLR-downstream target IFNβ. Interestingly, only LPS was able to reduce significantly the infiltration of MCF-7 into WT brain slices (Fig. 2A), while not affecting tumor cell invasion (Fig. 2B). Since MCF-7 infiltration was not affected in TRIF-deficient brain slices (Fig. 1D), we also analyzed the effects of LPS in these co-cultures, but observed no differences (Fig. 2C). This might indicate that TRIF does not contribute to the TLR4-mediated MG-assisted infiltration of tumor cells, but is required for the protective effect of LPS.

We then examined which cells respond most to LPS stimulation. Compared to other cellular components of the organotypic 3D ex vivo co-cultures, the metabolic activity of MG cells was significantly enhanced upon LPS stimulation (Fig. 2D). Thus, our previous [15] and current results indicate that LPS affects mainly MG and subsequent MG-assisted infiltration of MCF-7 into the brain slice. However, the contradictory observations that TLR4 stimulation as well as the absence of the receptor or its adapter MyD88 have the same effect on MCF-7 infiltration required further investigation. One explanation could be that low or high activation levels of TLR4/MyD88 signaling lead to significantly different expression levels of specific TLR4/MyD88 target genes, which in turn would affect the tumor infiltration capacity in opposing ways. Moreover, these dose-dependent effects on metastatic infiltration seem to be exclusive of the TLR4 signaling, since stimulation of other TLRs did not affect the infiltration of the breast cancer cells in our ex vivo model (Fig. 2A).

### NO as effector of TLR4 activation

To investigate this hypothesis, we first searched for effector molecules under the control of TLR4, which have significantly higher concentrations upon LPS stimulation in comparison to other TLR ligands. We identified nitric oxide (NO) as a potential candidate. NO is produced by the inducible NO synthase (iNOS), which is regulated by TLR4 besides other TLRs [19]. Thus, we first examined NO production by MG upon stimulation with the above-tested TLR agonists. The release of the short-lived NO was determined as nitrite accumulation. Our results confirmed a robust and high and moderate increase in nitrite after TLR4 or TLR2/1 stimulation, respectively. LPS-induced nitrite production was counteracted by the addition of the NO inhibitor Nω-nitro-L-arginine methyl ester (L-NAME). Moreover, we also identified a reduced nitrite concentration in the untreated control after addition of L-NAME, indicating a baseline production of NO by MG in mono-culture (Fig. 2E).

Next, we challenged the nitrite production by MG in MyD88- and TRIF-deficient situations. In both cases, nitrite release after LPS stimulation was not significantly increased compared to WT MG (Fig. 2F). However, the expression of the inducible NO synthase (*iNos*/*Nos2*) was significantly higher in all cases after LPS stimulation, albeit much more in WT MG (Fig. 2G). These data confirm NO as a downstream target of both TLR4 adapters MyD88 and TRIF.

In addition, we studied the impact of NO in our 3D organotypic brain-slice ex vivo co-culture model. Similar to the diminished MCF-7 infiltration upon LPS stimulation (Fig. 2A), the NO downstream target, 8-bromo-guanosine 3′, 5′-cyclic monophosphate (8-Br-cGMP), significantly reduced infiltration of MCF-7 into the brain slice (Fig. 2H). Furthermore, NO inhibition with L-NAME also resulted in a significant reduction of MCF-7 infiltration (Fig. 2H), similarly to the case in the TLR4- and MyD88-deficient microenvironments (Fig. 1D). These results reveal that NO has opposing effects in the brain parenchyma depending on its concentration: baseline NO fostered infiltration, whereas high concentrations prevented it.

### TLR4 signaling in syngeneic models

**Fig. 3:**
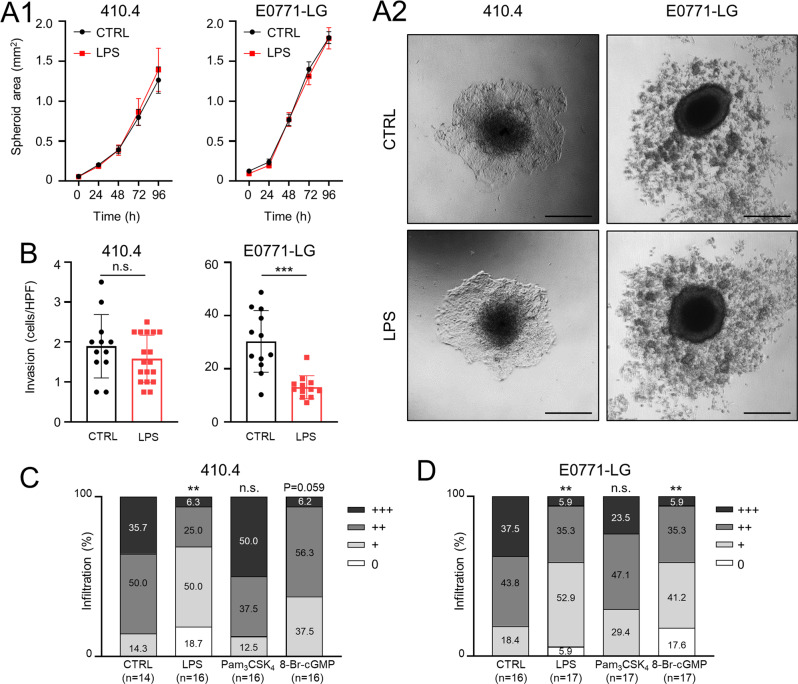
Effect of TLR activation on tumor cell growth, invasion and infiltration. **A** 3D tumor cell spheroids (410.4 left, E0771-LG right) formed by the hanging-drop method. (A1) The spheroid area (in mm^2^) upon treatment with LPS (1 µg/ml) is depicted compared to the control (CTRL; mean ± SD, *n* ≥ 3; two-way ANOVA followed by Sidak’s multiple comparisons test). (A2) Representative images of spheroid outgrowth after 96 h are illustrated. Scale bars represent 500 µm. **B** 410.4 (left) or E0771-LG (right) tumor cell invasion after PBS (CTRL) or LPS (1 µg/ml) stimulation. Data represent the average tumor cell number migrated per high power field (HPF) after 96 h (*n* ≥ 12; mean ± SD; n.s. = not significant, ***p < 0.001; unpaired *t*-test). Organotypic brain-slice ex vivo co-cultures with 410.4 (**C**) or E0771-LG (**D**) tumor cells and stimulated with LPS (1 µg/ml), Pam_3_CSK_4_ (1 µg/ml), or 8-Br-cGMP (10 µM). Data represent the percentage degree of tumor-cell infiltration into the brain slices (WT) after 96 hours (*n* ≥ 14; n.s. = not significant, **p < 0. 01; Mann-Whitney test).

Knowing that LPS stimulation reduces the infiltration of MCF-7 human breast cancer cells in 3D organotypic brain-slice ex vivo co-cultures, we next aimed to investigate the effects of TLR signaling activation in syngeneic models. For this purpose, we employed 410.4 (Balb/C) and E0771-LG (C57Bl/6) murine breast cancer cell lines. First, we tested the effects of LPS stimulation on tumor growth and invasion. While LPS did not affect tumor growth in any of the cell lines (Fig. 3A), it decreased the invasion of E0771-LG significantly (Fig. 3B). Similar to the results with the human MCF-7, only the stimulation of TLR4 (LPS) and not TLR2/1 (Pam_3_CSK_4_) significantly reduced the infiltration of murine 410.4 and E0771-LG cells in the organotypic brain-slice co-cultures (Fig. 3C, D). This is extremely important, taking into account that TLR1 expression significantly correlated with a poorer prognosis (Fig. 1A–C and Supplementary Fig. 1A). Moreover, stimulation with the NO downstream target, 8-Br-cGMP, significantly reduced infiltration of E0771-LG into the brain slice (Fig. 3D), as previously demonstrated for the human MCF-7 (Fig. 2H). 8-Br-cGMP also diminished infiltration of 410.4, albeit not significantly (Fig. 3C).

### TLR4 stimulation changes the histopathological growth pattern (HGP) of brain metastases at the MMPI

**Fig. 4:**
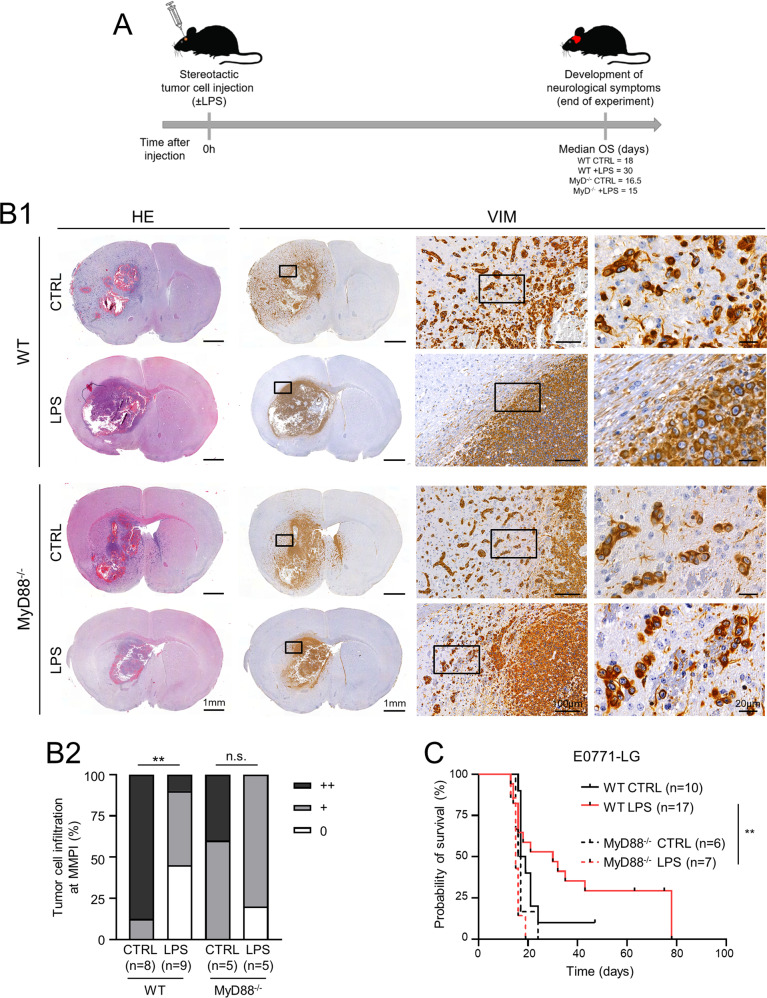
Impact of TLR4 activation via MyD88 on metastatic infiltration and survival. **A** Scheme depicting the timeline of the long-term in vivo experiment. Median OS of the different groups is indicated. **B** Characterization of E0771-LG infiltration at the MMPI. (B1) Representative images of histology (HE) and vimentin (VIM) immunohistochemistry (IHC) in coronal brain sections of wild-type (WT) or MyD88^−/−^ mice injected with E0771-LG tumor cells and stimulated either with PBS (CTRL) or with LPS (1 µg/ml). Higher magnification images portraying metastatic infiltration at the MMPI are also presented. (B2) Quantification of the degree of tumor cell infiltration at the MMPI is indicated as the percentage of animals with high (++), medium (+), or low (0) metastatic infiltration at the MMPI (*n* ≥ 5; n.s. = not significant, **p < 0.01; Mann-Whitney test). **C** Kaplan-Meier survival curves of WT (straight line) or MyD88^−/−^ (dashed line) mice injected with E0771-LG tumor cells and stimulated either with PBS (black) or with LPS (red) (*n* ≥ 6; **p < 0.01; log-rank test).

Encouraged by these results, we next aimed to investigate the effects of a single local LPS application during breast cancer colonization in an immunocompetent syngeneic model of brain metastasis. Since the MyD88^−/−^ mice had a C57Bl/6 background, we further worked with the C57Bl/6 murine breast cancer cell line E0771-LG for our in vivo experiments. We sought to analyze the effects of a single LPS treatment on overall survival (OS), immune infiltrate, and the histopathological growth pattern (HGP) of brain metastases at the MMPI. We previously demonstrated that the presence of tumor cells infiltrating the adjacent organ parenchyma (infiltrative HGP) usually correlates with a worse OS compared to well-demarcated metastases (displacing HGP) [20, 21]. Thus, we stereotactically injected the syngeneic E0771-LG cells into the mouse brain parenchyma and performed a single-point application of LPS simultaneously (day 0). The mice were sacrificed upon appearance of neurological symptoms as a sign of macro-metastatic growth (long-term experiment, Fig. 4A). Excitingly, this single LPS application on day 0 led to astonishing changes in the HGP of macro-metastases at the MMPI. Whereas untreated mice demonstrated high degrees of infiltration, mice undergoing LPS application revealed little to no infiltration by the end of the experiment (Fig. 4B). These effects were especially appreciable in WT mice. MyD88-deficient mice treated with LPS also demonstrated decreased infiltration at the MMPI; however, these differences were not significant (Fig. 4B).

Interestingly, the changes in the HGP of brain metastases at the MMPI were accompanied by changes in the OS: WT mice injected with E0771-LG and stimulated with LPS survived 30 days, while the median OS of untreated WT mice was only 18 days. Nevertheless, these differences in OS were not significant (*p* = 0.258; Fig. 4C). Importantly, LPS stimulation provided a survival advantage to WT mice compared to MyD88-deficient mice (median OS = 30 vs 15 days, respectively; p = 0.006; Fig. 4C). These data again indicate that MyD88 is necessary for the protective effect of LPS.

### TLR4 stimulation changes the macrophage/microglia (Mɸ/MG) response in brain metastasis

**Fig. 5:**
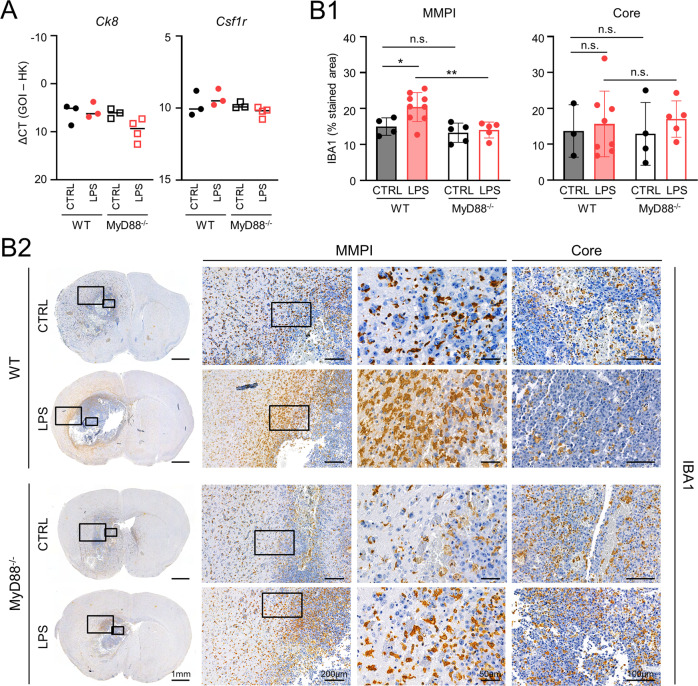
Impact of TLR4 activation on Mɸ/MG response in brain metastasis mouse models. **A** qPCR analysis of *Ck8* and *Csf1r* expression in brain metastases of wild-type (WT, dots) or MyD88^−/−^ (squares) mice injected with E0771-LG tumor cells and stimulated either with PBS (CTRL; black) or with LPS (red) (*n* ≥ 3; mean; one-way ANOVA followed by Sidak’s multiple comparisons). **B** Characterization of Mɸ/MG activation in brain metastases of wild-type (WT, full bars) or MyD88^−/−^ (empty bars) mice injected with E0771-LG tumor cells and stimulated either with PBS (CTRL; black) or with LPS (red). (B1) Quantification of IBA1 staining is indicated as the percentage of stained area in the respective brain region (*n* ≥ 4; mean ± SD; n.s. not significant, *p < 0.05, **p < 0.01; one-way ANOVA followed by Sidak’s multiple comparisons). (B2) IHC staining of activated Mɸ/MG (IBA1). Representative images of coronal brain sections and images of MMPI and tumor core at higher magnification are depicted.

Since the (protective) effects of TLR4 stimulation were lost in a MyD88-deficient environment, we next asked which (immune) cell types were mostly affected in the metastatic bulk tissue. Quantitative PCR (qPCR) analysis of the murine brain metastases revealed no significant differences in tumor load (*Ck8*), Mɸ/MG activation (*Csf1r*), or AS activation (*Gfap*) (Fig. 5A and Supplementary Fig. S2A) between WT and MyD88^−/−^ untreated or LPS-treated mice. However, owing to the astonishing changes observed in the HGP, we investigated whether we could detect localized differences in the immune compartment, in particular at the MMPI. We thus decided to stain the brain metastases with specific markers for Mɸ/MG (IBA1) and AS (GFAP) to reveal potential local differences. Strikingly, the single-point application of LPS at day 0 led to a significantly increased recruitment of Mɸ/MG to the MMPI (and not in the metastatic core) only in WT mice (Fig. 5B). Moreover, we did not observe obvious differences in the astrocytes´ compartment (Supplementary Fig. S2B), indicating that the main changes associated with TLR4 stimulation take place at the MMPI in the Mɸ/MG compartment.

### MyD88-target genes Il6 and Ccl2 contribute to metastatic colonization

**Fig. 6:**
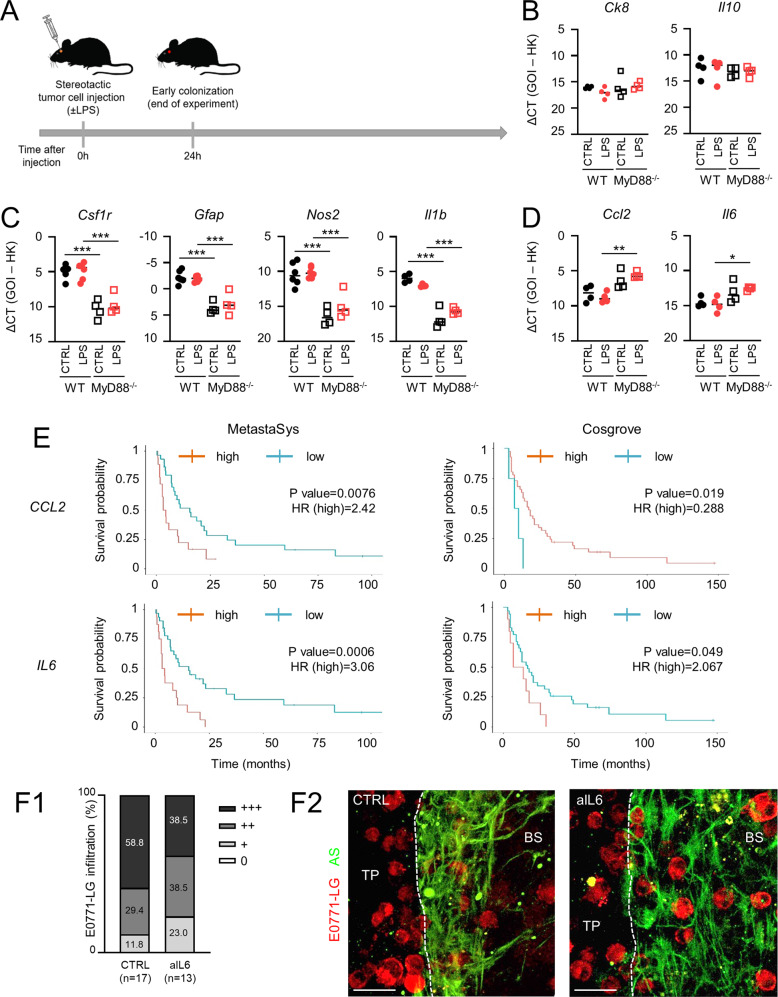
Target genes under the control of TLR4/MyD88. **A** Scheme depicting the timeline of the short-term in vivo experiments (early colonization). **B**–**D** qPCR analysis of the indicated genes in brain metastases of wild-type (WT, dots) or MyD88^−/−^ (squares) mice injected with E0771-LG tumor cells and stimulated either with PBS (CTRL; black) or with LPS (red). Tumors were isolated 24 h after tumor cell inoculation (*n* ≥ 4; mean; *p < 0.05, **p < 0.01, ***p < 0.001; one-way ANOVA followed by Sidak’s multiple comparisons). **E** Kaplan-Meier survival curves of BCBM patients in MetastaSys (left) or Cosgrove (right) cohorts stratified on the basis of *CCL2* (top) or *IL6* (bottom) expression (*P* value = log-rank test *p*-value; HR = hazard ratio). **F** Organotypic brain-slice ex vivo co-cultures with E0771-LG tumor cells stimulated with isotype control (CTRL) or anti-IL6 (1 µg/ml). Data represent the percentage degree of tumor cell infiltration into the brain slices (WT) after 96 hours (*n* ≥ 13; Mann-Whitney test). (F2) Representative pictures of the organotypic ex vivo co-cultures with E0771-LG cells (TP tumor plug, BS brain slice). E0771-LG tumor cells are depicted in red and AS (GFAP) are shown in green. Scale bars represent 30 µm.

Owing to the astonishing consequences of TLR4 stimulation during the long-term experiment, we sought to identify changes occurring at earlier time points potentially responsible for the MyD88-dependent protective effect of LPS. For this, we performed a short-term experiment, in which mice were sacrificed 24 h after tumor cell injection (Fig. 6A). qPCR analysis of mouse brain metastases revealed negligible metastatic burden (*Ck8*) at this early time point compared to the macro-metastases in the long-term experiment (ΔCt **=** 17 vs. 7, respectively) and did not differ among cohorts (Fig. 6B). Owing to the contribution of MyD88 to the protective effect of LPS, we investigated well-described MyD88 target genes, which are relevant during metastasis progression. Interestingly, not all MyD88 target genes were downregulated in MyD88-deficient mice. For example, the expression of the anti-inflammatory cytokine *Il10* remained unchanged (Fig. 6B), excluding a random downregulation of MyD88-downstream targets.

Due to differences seen in astrogliosis in the 3D organotypic ex vivo co-culture analysis (Fig. 1E, F and Supplementary Fig. S1B), we additionally investigated *Csfr1* and *Gfap* as markers for Mɸ/MG activation and astrogliosis, respectively. Comparable to the co-culture results, both *Csf1r* and *Gfap* were significantly decreased in the MyD88^-/-^ context at this early time point (Fig. 6C). Moreover, unlike *Il10*, we observed substantial changes in other MyD88-target genes in brain metastases of MyD88-deficient mice. For example, *Nos2* and *Il1b*, known to mediate the communication between Mɸ/MG and AS [22], were significantly downregulated in the MyD88-deficient microenvironment, regardless of LPS stimulation (Fig. 6C). This could explain the likewise significantly reduced expression of Mɸ/MG and AS marker genes.

Strikingly, *Ccl2* and *Il6*, usually associated with metastasis progression and poor prognosis [23, 24], were expressed greater in MyD88^−/−^ mice, reaching significance in the LPS-stimulated cohort (Fig. 6D), offering an explanation for the worse OS (Fig. 4C). To verify this hypothesis, we retrospectively analyzed the prognostic value of these two MyD88-target genes in the two human cohorts (MetastaSys and Cosgrove). While *CCL2* was associated with ambiguous outcomes, *IL6* significantly correlated with a poorer prognosis in both cohorts (Fig. 6E). Therefore, we concentrated on the pharmacological inhibition of IL6. However, IL6 blockade did not significantly impact E0771-LG infiltration into the brain slice (Fig. 6F). This could due to the fact that lymphocytes, which are also activated by IL6, are not present in the organotypic co-culture.

## Discussion

Our results reveal that *TLR1* and *TLR6* gene expression, both forming a heterodimer with *TLR2*, are associated with a worse OS of patients with BCBM. Furthermore, stimulation with Pam_3_CSK_4_, a TLR2/1 agonist, does not lead to a more effective anti-tumor activity in organotypic brain-slice ex vivo co-cultures of any breast cancer model. Thus activation of TLR2/1 does not represent a promising therapeutic strategy in BCBM.

However, blocking TLR2 on the receptor level could be a potential target in the CNS. This idea is supported by a glioma model in which TLR2 knockout resulted in reduced glioma growth. In this context, glioma-derived versican was identified as tumor-derived DAMP activating TLR2 on macrophages/microglia [25]. This result confirmed earlier findings, in which versican was also identified as tumor-derived DAMP in a lung-cancer model. In this model, tumor-derived versican was responsible for macrophage recruitment to the metastatic niche. Furthermore, TLR2 knockout reduced the metastatic capacity of this syngeneic lung-cancer model. Moreover, versican-mediated TLR2 activation led, among other effects, to upregulation of IL6. Signaling analysis of the tumor-derived versican-mediated TLR2 signaling identified TLR6 as necessary heterodimer and MyD88 as responsible TLR adapter [26].

While blocking on the TLR2 receptor-level could be a potential target, our results do not support any blockade of its adapter MyD88. Although the absence of MyD88 in the brain parenchyma led to reduced infiltration by tumor cells, it also reduced MG and AS activation in the organotypic brain-slice ex vivo co-cultures and in the short-term in vivo experiment. Most importantly, MyD88 knockout also revealed no survival benefit in the long-term syngeneic colonization model. Contrary to expectations, *Il6*, a well-known marker for tumor progression, was expressed greater in the MyD88^-/-^ group in the short-term experiment, in particular after LPS stimulation. *IL6* was also associated with poor prognosis in both BCBM data sets. Recently, IL6/PI3K signaling was described as an activator of disseminated tumor cells (DTCs) [27] and initial perivascular growing cancer cells are sensitive to dual PI3K/mTOR inhibition during brain colonization [28]. We previously identified PI3K as master regulator of MG and tumor-associated macrophages (TAMs) during BC metastatic colonization [16]. However, in contrast to the previously described PI3K-inhibition, anti-IL6 treatment did not reduce tumor cell infiltration significantly in our organotypic brain-slice ex vivo experiments. This is in line with the investigations of versican-induced lung-cancer metastasis, in which IL6 knockout caused only small differences in survival [26]. These findings suggest that PI3K is a more promising target than IL6 or MyD88 in BCBM.

Finally, the results of TLR4 are also ambiguous, like the MyD88 data. This is already evident from the survival analyses of the two BCBM data sets. In these analyses, TLR4 is the only TLR, which has an opposite significant prognostic association with survival. There are already controversial reports of reduced tumor growth and improved survival in TLR4-deficient mice [29–31]. On the contrary, TLR4 activation also leads to the production of IFNβ by tumor cells, contributing to an anti-tumor immune response [32]. These studies indicate that TLR4 signaling and some TLR4 target genes have dual roles during metastatic outgrowth. Our data demonstrate that a robust glial defense may only be achieved upon activation of both MyD88 and TRIF. The fact that TLR4 is the only TLR to signal via both adapters makes its stimulation an attractive therapeutic approach. Moreover, LPS led to the most robust NO induction, which is well-known to have a tumoricidal effect at high concentrations [33–35]. On the contrary, low NO concentrations seem to be necessary for metastatic infiltration at the MMPI, which is also supported by previous findings [36].

Taking our in vitro and in vivo results together, we demonstrate here that a robust (local) activation of TLR4 at colonization onset strengthens the local defense during further metastatic outgrowth. This effect is most likely visible at the MMPI, at which a single intralesional LPS treatment at the beginning led to the formation of a compact macrophage wall and a significant reduction in the infiltration of metastatic cells into the adjacent tissue at the end of the experiment. This improved containment of the metastatic bulk required robust local activation of both TLR adapters MyD88 and TRIF. At least at this point, one might remember the earlier clinical studies by William Coley, who also applied a PAMP cocktail comprising bacterial components intralesionally to treat cancer [37]. In view of Coley´s early clinical studies and our results, additional LPS treatment on the brain cavity wall after metastasis resection might be a valid study concept to strengthen the local defense system for example in oligo-metastasized disease of BC patients.

## Materials and methods

### Cell culture and transfection

MCF-7 cells (DSMZ GmbH, Braunschweig, Germany) were grown on RPMI-1640 medium (PAA Laboratories Inc., Cölbe, Germany) supplemented with 10% fetal calf serum (FCS, Invitrogen, Karlsruhe, Germany). E0771-LG and 410.4 cells were kindly provided by Profs. J. Pollard and. F. Balkwill (London, UK), respectively, and grown on DMEM 10% FCS. Primary microglia and astrocytes were prepared as previously described [38]. Cells were regularly tested for mycoplasma.

Cells were stably transfected with the plasmid pEGFP-actin (Clontech, Heidelberg, Germany) and mammalian Turbo GFP vector (FP512, Evrogen Inc., Heidelberg, Germany) using a Nanofectin kit (PAA, Cölbe, Germany), or retrovirally transfected with a TdTomato plasmid (pLib_E1A_mtdTomato-2A-puro, Clontech). Selection was achieved using geneticin (Roche, Basel, Switzerland) or puromycin (Sigma, Munich, Germany). Cells were sorted regularly (BD FACS Aria II, Heidelberg, Germany).

### Reagents

Pam_3_CSK_4_ and LPS were purchased from Enzo Life Sciences/Alexis (Lörrach, Germany). Poly(A:U) and 8-bromoguanosine-3′, 5′-cyclic monophosphate (8-Br-cGMP) were purchased from Sigma. Mouse interferon-β (IFNβ) was sourced from PBL Biomedical Laboratories (Piscataway, NJ, USA). Nitric oxide (NO) synthase inhibitor, Nω-nitro-L-arginine methyl ester (L-NAME) was obtained from Cayman Chemical (Hamburg, Germany). Anti-interleukin-6 (aIL6) monoclonal antibody (clone MP5-20F3, cat#BE0046) and the isotype control (rat IgG1, cat#BE0088) were purchased from Biozol (Eching, Germany).

### Animal models

Animals were bred in the University Medical Center Göttingen (Germany) animal facility and maintained under specific pathogen-free conditions. TLR4 [39], MyD88 [40], and TRIF [41] knockout (KO) mice were kindly provided by Prof. U. K. Hanisch (Göttingen, Germany). All animal work was approved by the Government of Lower Saxony (approval no. 33.4-42502-04-13/1266).

Stereotaxic intracortical injection was performed as described previously [16]. Briefly, 12-week-old female mice were injected stereotactically with tumor cells (3 µl) and 1 µl PBS or LPS (1 µg/ml) and sacrificed either 24 hours later (short-term experiment) or upon development of neurological symptoms (long-term experiment). Development of neurological symptoms was evaluated in a blind manner. No randomization was used.

### Organotypic brain-slice ex vivo co-culture system

Organotypic brain-slice ex vivo co-cultures were performed as described previously [15, 17, 42]. The grade of tumor cell infiltration was evaluated in a blind manner.

### Immunofluorescence (IF) staining

IF staining of astrocytes and microglia in organotypic ex vivo co-cultures was performed as described previously [15, 17, 42]. Image J software (Image J 1.43 f [43]) was used to quantify microglia cells or astrocyte protrusions.

To detect iNOS expression, 2 × 10^5^ microglial cells were seeded on a coverslip and cultured with or without LPS for 24 hours followed by 4% PFA fixation. Polyclonal anti-iNOS antibody (Merck Millipore, Darmstadt, Germany, cat#ABN26) followed by anti-rabbit FITC (Sigma, cat#AP132F) staining was performed at RT for 1 hour each.

### Cell viability assay

Cells were cultured with PBS or LPS for 24 hours. Metabolic activity was analyzed by measuring 2,3-diphenyl-5-methyltetrazolium chloride (MTT; Sigma) conversion according to standard procedures.

### NO measurements

NO synthesis was measured in culture supernatants as the end product, i.e., nitrite, using Griess reagent (Merck) as described [19].

### Reverse transcription and quantitative real-time polymerase chain reaction (qPCR)

RNA isolation, cDNA synthesis and qPCR were performed as described [44]. For primer sequences, refer to Supplementary Table 2.

### 3D spheroid cultures (hanging drop assay)

Drops of 25 µl of medium containing 250 tumor cells were placed on the inside of the lid of a 100-mm plate filled with PBS to create a hydration chamber. Next, the lid was carefully inverted 180° and the hanging drops were incubated for 72 h at 37 °C. Spheroids were transferred into 24-well plates (one spheroid per well) containing cell-culture medium with either PBS or LPS (1 µg/ml) and incubated up to 96 hours at 37 °C. Spheroid outgrowth (mm^2^) was analyzed with ImageJ.

### Boyden Chamber (invasion assay)

The degree of tumor cell invasion was measured using a Boyden chamber assay as described previously [15].

### Histology and immunohistochemistry (IHC) of murine brain metastasis

Hematoxylin-eosin (HE) and IHC of murine brain metastasis were performed as described [16]. The degree of tumor cell infiltration at the MMPI was assessed by an experienced observer in a blind manner using the following scoring system: 0 (no infiltration), + (low infiltration), ++ (high infiltration). Microglia and astrocytes were quantified in triplicate images of selected regions of interest (ROIs) using ImageJ software. Briefly, the RGB image channels were split using color deconvolution. The appropriate channels (brown for IBA1, red for GFAP) were selected and previously established thresholds (defined by pre-analysis) were applied. Finally, the percentage of stained area was measured.

### Patient cohorts and RNA sequencing

RNA sequencing (RNA-Seq) data were collected for two BCBM patient cohorts: MetastaSys (*n* = 48) and Cosgrove et al. [45] (*n* = 45). For detailed clinical data, see Supplementary Table 3.

MetastaSys cohort: Human CNS material was obtained from the archives of the Institute of Neuropathology (Göttingen) and the Department of Neuropathology (Regensburg) in accordance with the ethics review board of the University Medical Center Göttingen (decision no. 24/10/05). All subjects provided informed consent. RNA extraction, library preparation, amplification, sequencing, and RNA-Seq data pre-processing were performed as described [20]. RNA-Seq data of 17 BCBM samples were uploaded to the GEO repository under the identifier GSE196519. RNA-Seq raw count data of 31 (GSE161865) and 17 (GSE196519) BCBM samples were normalized using the trimmed mean of M-values (TMM) method in the Bioconductor package ‘edgeR’ (v3.36.0, https://bioconductor.org/packages/edgeR/) [21], and log2-normalized counts (log2 CPM TMM) were obtained.

Cosgrove cohort: Normalized RNA-Seq count data (log2 CPM TMM) were downloaded from the NCBI Gene Expression Omnibus website (GSE184869). Clinical data were downloaded from [10.6084/m9.figshare.16685680.v1].

### Clustering and heatmap

Gene expression levels were visualized using the R package ‘pheatmap’ (v4.2.1, https://CRAN.R-project.org/package=pheatmap) with the Ward D2 linkage algorithm chosen for clustering and standardization of expression levels of each gene in different samples.

### Survival analysis

Two groups (high/low) were defined based on expression levels for individual genes of interest using the maximally selected rank statistics (maxstat) method in the R package ‘survminer’ (v0.4.9, https://CRAN.R-project.org/package=survminer). The surv_cutpoint function in the survminer package was used to determine the optimal cut-off values for the assessed genes.

The log-rank test was utilized to assess different survival rates across the groups. Significance p-values and hazard ratios (HR) were calculated using the R package ‘survival’ (v3.3.1, https://CRAN.R-project.org/package=survival). For detailed survival analysis data, see Supplementary Table 1.

### Statistics

Samples represent biological replicates (mean values of ≥2 technical replicates). Groups with similar variance were compared. Statistical differences were analyzed using GraphPad Prism software version 9 (GraphPad, San Diego, CA). *P* **<** 0.05 was regarded as statistically significant. For animal studies, sample size estimate was calculated with G-Power (Fisher Exact Test, Power 80%, β-error 20%).

## Supplementary information

Supplementary Information

Supplementary Figure S1

Supplementary Figure S2

## Data Availability

R code is available upon request.
