# Supplementary material for: Intralesional TLR4 agonist treatment strengthens the organ defense against colonizing cancer cells in the brain
Source: Oncogene. 2022 Oct 12;41(46):5008–19. doi: 10.1038/s41388-022-02496-3 (PMC9652147; doi:10.1038/s41388-022-02496-3)
Supplement: Supplementary file 1 — Supplementary Information [file 41388_2022_2496_MOESM1_ESM.docx]

Supplementary Figure Legends

Supplementary Figure S1: Clinical relevance of TLR expression in human brain metastases and contribution of TLR4 to tumor cell infiltration (cont.). (A) Kaplan-Meier survival curves of BCBM patients in MetastaSys (left) or Cosgrove (right) cohorts stratified on the basis of the expression of the indicated genes (P=log-rank test p-value; HR=hazard ratio). (B) Representative pictures (left) and quantification (right) of MG or AS activation are illustrated. The area covered with MG inside the brain slice (BS) was quantified (upper row). The length of the astrocytic protrusions (in µm) was measured at the MMPI (lower row) (n≥12; mean±SEM; ***p<0.001; unpaired t-test).

Supplementary Figure S2: Impact of TLR4 activation on AS response in brain metastasis mouse models. (A) Quantitative RT-PCR analysis of *Gfap* expression in brain metastases of wild-type (WT, dots) or MyD88^-/-^ (squares) mice injected with E0771-LG tumor cells and stimulated either with PBS (CTRL; black) or with LPS (red) (n≥3; mean; one-way ANOVA followed by Sidak’s multiple comparisons). (B) Characterization of AS activation in brain metastases of wild-type (WT, full bars) or MyD88^-/-^ (empty bars) mice injected with E0771-LG tumor cells and stimulated either with PBS (CTRL; black) or with LPS (red). (B1) Quantification of GFAP staining is indicated as the percentage of stained area in the MMPI (n≥4; mean±SD; one-way ANOVA followed by Sidak’s multiple comparisons). (B2) Immunohistochemical (IHC) staining of activated astrocytes (GFAP). Representative images of coronal brain sections and images of MMPI at higher magnification are depicted.

Supplementary Tables

**Supplementary Table 1: Survival analysis**

*MetastaSys cohort (n=48)*

|  | **Maximally selected rank statistics (MaxStat)** | **Kaplan-Meier logrank test** | **Cox proportional hazard model** | |
| --- | --- | --- | --- | --- |
| Gene name | Optimal cutpoint  (high/low groups) | p.value | HR (high) | p.value |
| TLR1 | 2.33 | 0.016* | 2.31 | 0.0192* |
| TLR2 | 3.85 | 0.052 | 0.532 | 0.0561 |
| TLR3 | 1.97 | 0.12 | 1.65 | 0.125 |
| TLR4 | 3.32 | 0.0203* | 2.14 | 0.233 |
| TLR5 | 3.71 | 0.2 | 1.56 | 0.204 |
| TLR6 | 1.88 | 0.0107* | 2.42 | 0.0134* |
| TLR7 | 2.11 | 0.049* | 1.91 | 0.0528 |
| TLR8 | 0.668 | 0.37 | 0.751 | 0.376 |
| TLR9 | 0.218 | 0.21 | 0.672 | 0.212 |
| MYD88 | 5.97 | 0.245 | 1.69 | 0.247 |
| TICAM1 | 4.57 | 0.0959 | 0.484 | 0.103 |
| CD14 | 5.95 | 0.066 | 0.542 | 0.0694 |
| CD40 | 3.53 | 0.021* | 2.16 | 0.024* |
| CD80 | 1.15 | 0.045* | 2.08 | 0.0499* |
| CD86 | 2.95 | 0.22 | 0.676 | 0.676 |
| CCL2 | 4.46 | 0.00731* | 2.42 | 0.00941* |
| IL6 | 0.854 | 0.00067* | 3.06 | 0.00122* |

*Cosgrove et al. cohort (n=45)*

|  | **Maximally selected rank statistics (MaxStat)** | **Kaplan-Meier logrank test** | **Cox proportional hazard model** | |
| --- | --- | --- | --- | --- |
| Gene name | Optimal cutpoint  (high/low groups) | p.value | HR(high) | p.value |
| TLR1 | 5.48 | 0.018* | 3.14 | 0.0229* |
| TLR2 | 2.44 | 0.12 | 1.87 | 0.12 |
| TLR3 | 4.02 | 0.22 | 0.485 | 0.23 |
| TLR4 | 4.82 | 0.0092* | 0.363 | 0.0125* |
| TLR5 | 4.41 | 0.052 | 1.89 | 0.0542 |
| TLR6 | 4.89 | 0.018* | 3.14 | 0.0229* |
| TLR7 | 2.12 | 0.078 | 0.577 | 0.0906 |
| TLR8 | 2.1 | 0.074 | 0.429 | 0.0837 |
| TLR9 | filtered out in Cosgrove et al. 2021 | | | |
| MYD88 | 4.74 | 0.19 | 0.651 | 0.197 |
| TICAM1 | 2.26 | 0.013* | 2.91 | 0.0175* |
| CD14 | 3.05 | 0.018* | 0.457 | 0.0226* |
| CD40 | 3.63 | 0.43 | 0.663 | 0.443 |
| CD80 | 0.774 | 0.17 | 1.74 | 0.171 |
| CD86 | 1.44 | 0.11 | 0.582 | 0.122 |
| CCL2 | 1.11 | 0.019* | 0.288 | 0.0266* |
| IL6 | 0.641 | 0.049* | 2.07 | 0.055 |

**Supplementary Table 2: Primer sequences**

| **Gene** | **Forward Primer (5🡪3)** | **Reverse Primer (5🡪3)** | **Amplicon Size (bp)** |
| --- | --- | --- | --- |
| Ccl2 | GGAGAGCTACAAGAGGATCAC | GTATGTCTGGACCCATTCCT | 114 |
| *Ck8* | ATGAACAAGGTGGAACTAGAG | ATCTCCTCTTCATGGATCTG | 83 |
| *Csf1r* | CACCATCCACTTGTATGTC | CTCAACCACTGTCACCTC | 72 |
| *Gfap* | AACCTGGCTGCGTATAGAC | CCAGCGATTCAACCTTTCTC | 78 |
| *Il1b* | GTAATGAAAGACGGCACACC | ACTCTGCAGACTCAAACTCC | 135 |
| *Il6* | GATGGATGCTACCAAACTG | CCAGGTAGCTATGGTACTC | 84 |
| *Il10* | GAAGACAATAACTGCACCCA | CATTAAGGAGTCGGTTAGCAG | 137 |
| *Nos2* | GTCTTGCAAGCTGATGGTCA | ACCACTCGTACTTGGGATGC | 94 |
| *Pgk1 (HK)* | GAAGGGAAGGGAAAAGATGC | GCTATGGGCTCGGTGTGC | 137 |
| *18S rRNA (HK)* | GTAACCCGTTGAACCCCATT | CCATCCAATCGGTAGTAGCG | 151 |

* Ccl2=chemokine (C-C motif) ligand 2; Ck8=cytokeratin 8; Csfr1=colony stimulating factor 1 receptor; Gfap=glial fibrillary acidic protein; Il1b=interleukin 1 beta; Il6=interleukin 6; Il10=interleukin 10; Nos2=nitric oxide synthase 2, inducible; Pgk1=phosphoglycerate kinase 1; 18S rRNA=18S ribosomal RNA; HK=house keeping gene.

**Supplementary Table 3: Patient clinical data**

*MetastaSys cohort (n=48)*

| **Clinical subtype** | **ER+/HER2−** | **HER2+** | **TNBC** |
| --- | --- | --- | --- |
| **Number of patients** | 13 | 21 | 14 |
| **Age at surgery (median [min, max])** | 60 [38, 78] | 54 [32,77] | 66 [43, 76] |
| **Age group (%)** |  | | |
| **<40** | 1 (7.7) | 4 (19) | 0 (0) |
| **40–59** | 5 (38.5) | 10 (47.6) | 6 (42.9) |
| **≥60** | 7 (53.8) | 7 (33.3) | 8 (57.1) |
| **Vital status (%)** |  | | |
| **Alive** | 2 (15.4) | 5 (23.8) | 1 (7.1) |
| **Dead** | 11 (84.6) | 16 (76.2) | 13 (92.9) |
| **SPBM***  **(median [min, max])** | 6.8 [1.5, 83.1] | 10.2 [1.4, 108.8] | 4 [0.3, 20.8] |

*SPBM = survival post brain metastasis (time from brain metastases surgery to death or follow up) in months.

*Cosgrove et al. cohort (n=45)*

| **Clinical subtype** | **ER+/HER2−** | **HER2+** | **TNBC** |
| --- | --- | --- | --- |
| **Number of patients** | 7 | 23 | 15 |
| **Age at diagnosis (median [min, max])** | 58 [40, 62] | 51 [26, 67] | 44 [26, 66] |
| **Age group (%)** |  | | |
| **<40** | 0 (0.0) | 4 (17.4) | 5 (33.3) |
| **40-59** | 4 (57.1) | 15 (65.2) | 8 (53.3) |
| **≥60** | 3 (42.9) | 4 (17.4) | 2 (13.3) |
| **Vital status (%)** |  | | |
| **Alive** | 0 (0.0) | 1 (4.4) | 3 (20.0) |
| **Dead** | 7 (100.0) | 22 (95.6) | 12 (80.0) |
| **SPBM***  **(median [min, max])** | 9 [5, 33] | 18 [3, 74] | 14 [3, 147] |

*SPBM = survival post brain metastasis (time from brain metastases surgery to death or follow up) in months.
