# Supplementary figures and images for: Intralesional TLR4 agonist treatment strengthens the organ defense against colonizing cancer cells in the brain
Source: Oncogene. 2022 Oct 12;41(46):5008–19. doi: 10.1038/s41388-022-02496-3 (PMC9652147; doi:10.1038/s41388-022-02496-3)

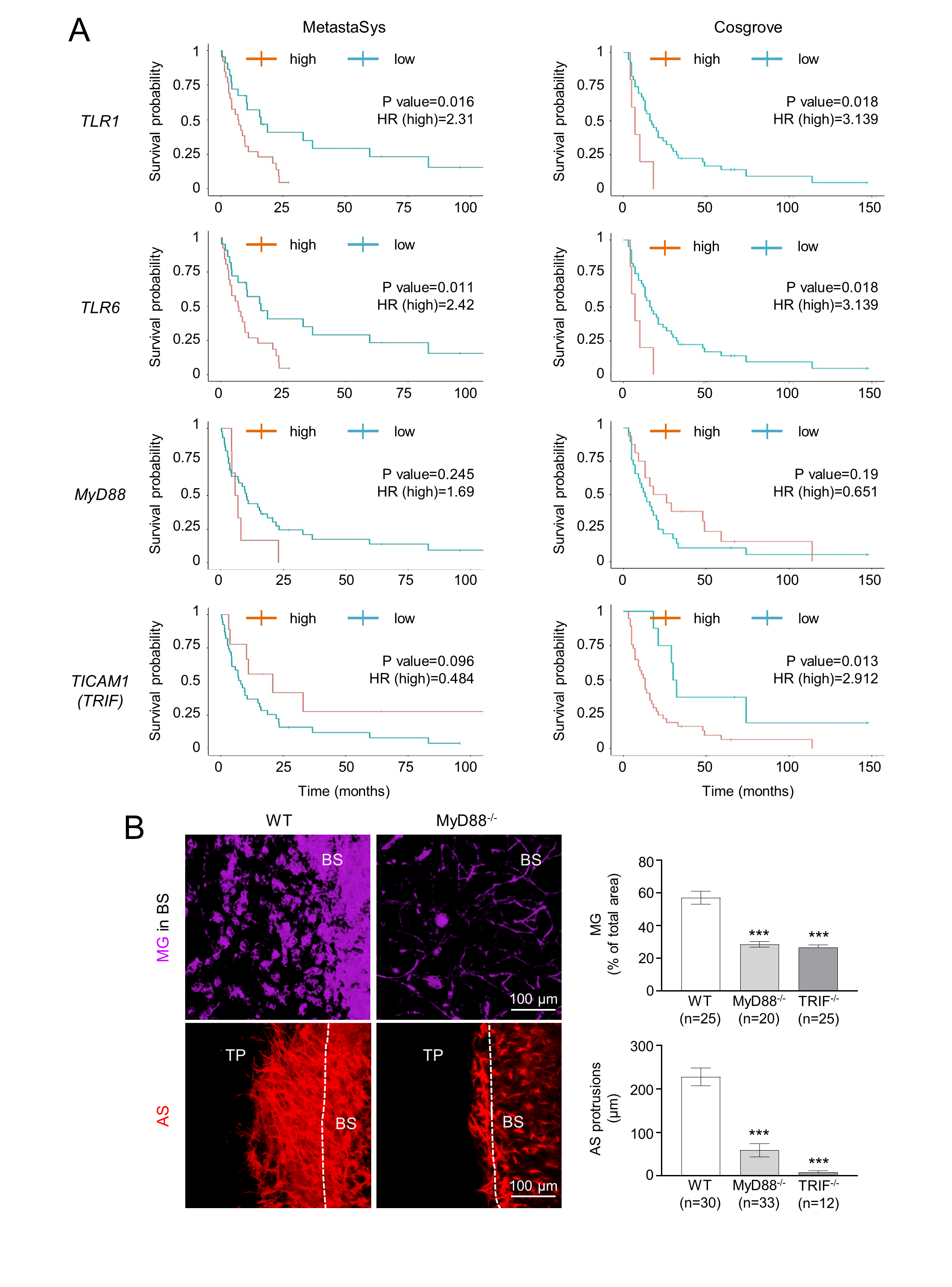

Supplement: Supplementary file 2 — Supplementary Figure S1 [file 41388_2022_2496_MOESM2_ESM.tif]

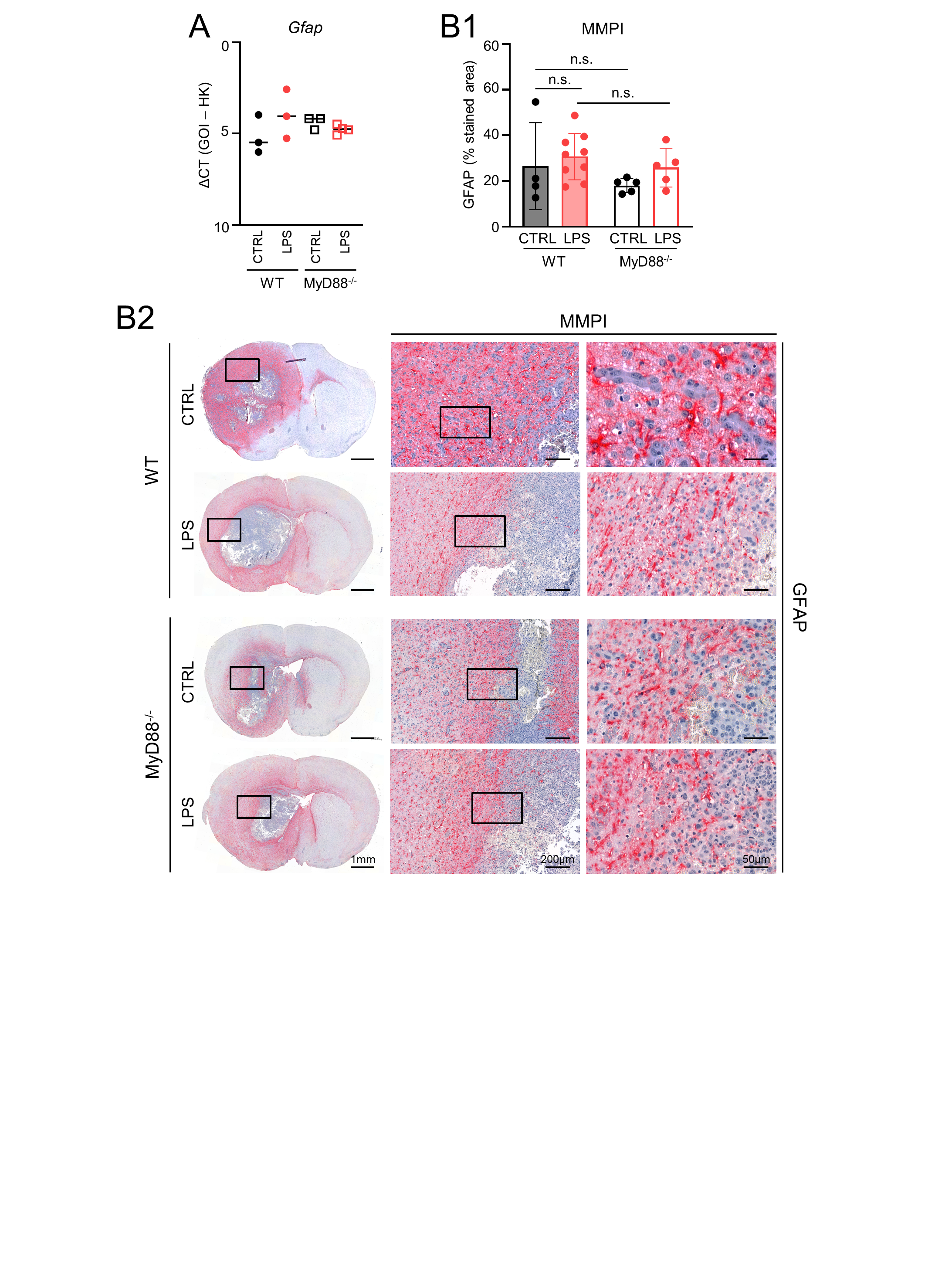

Supplement: Supplementary file 3 — Supplementary Figure S2 [file 41388_2022_2496_MOESM3_ESM.tif]
